# Palliative management of malignant duodenocolic fistula: Case report on endoscopic duodenal stent placement and its clinical implications

**DOI:** 10.1016/j.ijscr.2025.110918

**Published:** 2025-01-22

**Authors:** Guro Bjørnå, Mai-Britt Worm Ørntoft, Claudia Jaensch

**Affiliations:** a Surgical Department, Gødstrup Hospital, Denmark; b Surgical Research Department, Gødstrup Hospital and NIDO, Centre for Research and Education, Herning, Denmark

**Keywords:** Malignant duodenocolic fistula, Endoscopic stent, Colorectal cancer, Palliative treatment, Minimally invasive surgery

## Abstract

**Introduction and importance:**

Malignant duodenocolic fistulas are a rare but serious complication of advanced colorectal cancer. With the growing elderly population and increasing incidence of advanced colorectal cancer, there is a pressing need to explore palliative alternatives to complete resection, especially when a patient's overall health precludes extensive surgery.

**Case presentation:**

This case report presents a palliative approach involving luminal stent placement via gastroscopy in a patient with non-resectable, locally invasive colorectal cancer, resulting in a malignant duodenocolic fistula.

**Clinical discussion:**

We discuss different palliative treatment strategies against malignant duodenocolic fistulas, including endoscopic luminal stent placement and specific technical aspects of this procedure, highlighting factors that may contribute to a successful clinical outcome.

**Conclusion:**

Endoscopic stent placement can represent a minimally invasive palliative strategy to provide symptom relief in a patient with advanced colorectal cancer. Treatment strategy should be considered on a individual basis and in close consultation with the patient.

## Highlights

• Malignant duodenocolic fistulas are a serious complication of advanced colorectal cancer.
• Advanced colorectal cancer requires palliative alternatives to complete resection.
• Endoscopic stent insertion is a minimally invasive and safe palliative treatment option.
• Stent insertion offers several significant benefits and can improve quality of life.
• Correct positioning, anchoring and measurement of the stent might improve clinical outcome.

## 1 Introduction

Intestinal fistulas are most often seen as post-operative complications and secondary outcomes of iatrogenic procedures or trauma [1]. A minority occur because of severe inflammatory activity, as seen in patients suffering from e.g., inflammatory bowel disease, diverticulitis, pancreatitis, or cholecystitis [[2], [3], [4]]. Duodenal fistulas are rare and arise primarily due to duodenal ulcers or gallbladder stone migration [5].

A malignant intestinal fistula is a rare and potentially life-threatening condition. Most often, the fistula will manifest as an enterocutaneous fistula in colorectal cancer, permitting feces to exit through the skin, due to an abnormal connection between the intestine and through the abdominal wall. A rarer and more challenging presentation is a malignant entero-entero fistula, between two otherwise separated intestinal compartments. Especially malignant duodenocolic fistula pose a challenge, as the nutritional bolus will bypass the entire small bowel [6]. Malignant duodenocolic fistulas most often arise from colon cancer in the transverse colon or the right colonic flexure and present a surgical challenge, as resection requires extensive surgery, and implies grave post-operative complications. Furthermore, significant nutritional deficits are often present at the time of diagnosis, compromising the patient's ability to tolerate radical surgery. Often, this excludes both curative surgery in terms of en-bloc resection of the tumor mass, and extensive surgery such as establishment of a tube jejunostomy and loop ileostomy to facilitate feeding and provide symptom relief [7].

With the growing elderly population and increasing incidence of advanced colorectal cancer [8], there is a pressing need to explore palliative alternatives in unresectable and/or inoperable patients. This case-report describes the clinical presentation and palliative surgical treatment of a malignant duodenocolic fistula, using luminal stent placement via gastroscopy. As a malignant duodenocolic fistula is a rare presentation [7], information on clinical outcomes for treatment alternatives to radical surgery is important to assess individually when considering treatment possibilities.

This case-report was reported in line with the Surgical Case Reports (SCARE) guidelines [9].

## 2 Case description

**Fig. 1:**
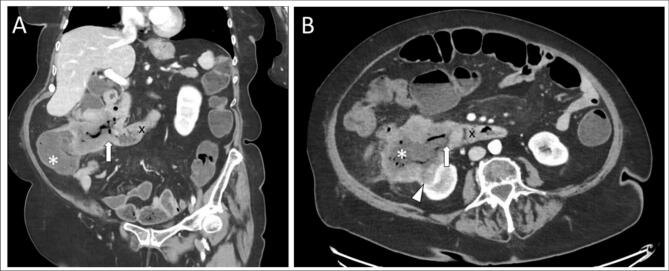
Initial coronal (A) and axial (B) computed tomography (CT) scan of the abdomen, with a tumor arising from the ascending colon (*), with local invasion of the right kidney (white arrowhead) and a malignant duodenocolic fistula (thick white arrow), connecting the ascending colon with the duodenal tract (x).

An 82-year-old woman, without previous history of abdominal surgery, was admitted because of sudden debut of excessive diarrhea and vomiting over a period of one and a half week. Initially, the tentative diagnosis was gastroenteritis. The patient had been treated with antibiotics (Ciprofloxacin) without clinical improvement. Diffuse abdominal pain and vertigo were present at admission. Biochemical evaluation revealed elevated infection markers (C-reactive protein: 91.9 mg/l, Leucocyte count: 16 × 10^9^/l, dominant neutrophil count, and thrombocytosis) and moderate anemia (hemoglobin: 5.9 mmol/l). Supplementary paraclinical tests, including feces samples and a computed tomography (CT) scan of the abdomen, were performed. The CT-scan showed a locally invasive tumor in the ascending colon with a fistula connecting the large intestine with the duodenal tract (Fig. 1).

A cancer care pathway was initiated, including a CT scan of the thorax, a colonoscopy with biopsies, and a gastroscopy, which confirmed a fistula between the descending part of the duodenum and the ascending colon. Biopsies histologically revealed colonic adenocarcinoma. Based on the American Joint Committee on Cancer (AJCC) TNM (Tumor, Node, Metastasis)-staging system [10], cancer stage was T4N1M0. Treatment options were discussed at the Multidisciplinary team conference (MDT), and it was concluded that the patient was inoperable. Likewise, palliative chemotherapy was not considered feasible due to ongoing Methotrexate-treatment of seropositive arthritis.

Alternative palliative treatment strategies were discussed. As the patient was considered operable for a minimally invasive procedure, the possibility of endoscopically insertion of a fully covered stent in the duodenal tract was discussed; A covered stent would theoretically block the nutritional bolus overflow to the colon through the fistula, allowing reestablishment of the intestinal tenue. This could subsequently improve the nutritional uptake, reduce the symptom burden, and thereby increase the quality of life and overall survival for the patient. However, endoscopic stenting of a duodenocolic fistula was experimental, scantly described in literature [11]. Still, it was decided to be the best treatment option available.

**Fig. 2:**
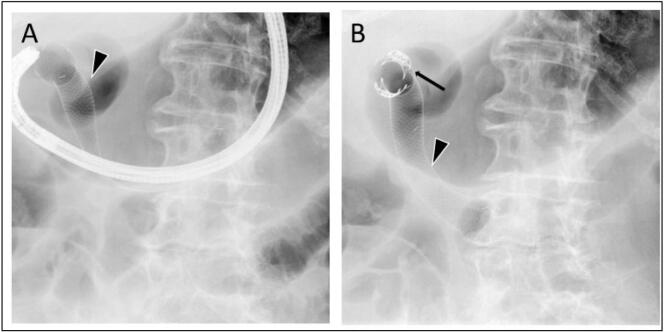
A: Intraoperative X-ray during stent placement by gastroscopy, securing correct insertion and placement of the Niti-S fully covered duodenal stent (black arrowhead) in relation to the fistula. B: X-ray after the procedure was finished, demonstrating the attachment of the stent, secured by two OTSC clips (thin black arrow) in the proximal end of the stent.

A gastroscopy was performed by a consultant in gastrointestinal surgery using a colonoscope (EVIS EXERA II 180, Olympus Corporation, Japan), and a 20 cm Niti-S fully covered duodenal stent (Taewoong Medical Co., Ltd., South Korea) was inserted under X-ray guidance. A colonoscope rather than gastroscope was chosen to ensure the ligament of Treitz could be reached during the procedure. Distal release of the stent was initiated at the level of the Treitz ligament, followed by continuous retraction past the 3 cm wide opening of the fistula, thereby fully covering the perforation, with the proximal end of the stent located in the pylorus. The stent was secured by two OTSC clips (Ovesco Endoscopy AG, Germany) in the oral end (Fig. 2).

**Fig. 3:**
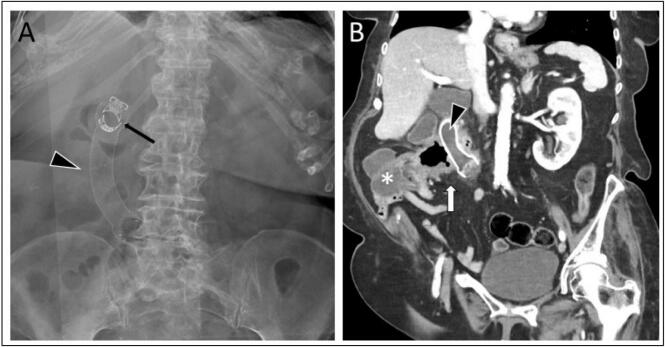
A: Conventional X-ray in anterior-posterior (AP) projection. B: Computed tomography (CT) scan of the abdomen. Diagnostic imaging performed three weeks after the endoscopic placement of a fully covered duodenal stent. The stent is correctly positioned (black arrowhead), and still fixated orally by the two OTSC slips (thin black arrow), hereby covering the opening of the malignant fistula (thick white arrow) created by the ascending colon tumor (*).

Resumption of a regular diet was possible immediately after the surgical procedure, with daily Bristol scale 4–6 bowel movements until the patient was discharged to a palliative care unit on day four. However, the patient experienced frequent episodes of diarrhea lasting several days after discharge. Due to suspected stent displacement, CT and X-ray examinations were done three weeks after the procedure. Here, the stent was found to be correctly positioned (Fig. 3). Symptoms were attributed to retrograde passage of bowel contents beyond the distal end of the stent. The patient died at home, four weeks after the procedure.

## 3 Discussion

Malignant duodenocolic fistulas are extremely rare but present a grave burden for the patient. A review from 1947 reported two duodenocolic fistulas in 1400 patients with carcinomas in the right colon, corresponding to an incidence of 0,14% [12]. A total of 70 cases have been reported since its first description by Haldane in 1862 [7]. Median overall survival is not well described in literature and varies considerably. In a review overlooking 8 cases of malignant duodenocolic fistulas between 1975 and 2005, median survival of 20 months were described, ranging between 1 and 150 months [4].

The only curative treatment for a malignant duodenocolic fistula is radical surgery, involving en-bloc right hemicolectomy combined with duodenectomy or pancreaticoduodenectomy /Whipple procedure [13]. However, resectability depends on the extent of the tumor and the presence of metastatic disease. Further, operability is often compromised due to high age and performance status, thus in most cases palliative treatment is the only reasonable option. Several palliative strategies have been reported. Spontaneous fistula closure after chemoradiation has been described [14]. Also, symptom management with octreotide treatment might be advantageous [15]. Surgical alternatives to complete resection include operative bypass techniques [16], or endoscopic stent placement to restore the integrity of the gastrointestinal wall [11] like in the present case*.*

Palliative treatment with stent insertion offers several significant benefits: A stent restores the intestinal tenue, allowing absorption of essential nutrients and fluids, while alleviating symptoms such as vomiting, chronic diarrhea, and abdominal pain, which otherwise rapidly compromises the patient's resources, quality of life and overall survival. Closing the communicating fistula also reduces inflammation of the colonic mucosa by gastric acid and bile salt exposure, and prevents bacterial translocation to the duodenum [17]. Additionally, it ensures that orally administered medications are properly absorbed in the small intestine, preventing them from bypassing this crucial area and losing their effectiveness. This is particularly important in managing symptoms like pain, nausea, and anxiety in advanced cancer patients. Without effective intestinal absorption, this could necessitate more intensive nursing care, potentially compromising the patient's autonomy and self-efficacy.

Endoscopic stent insertion is a minimally invasive treatment for a patient group characterized by poor performance status, comorbidities, frailty, and limited resources. Nevertheless, a feared complication is stent migration, as this will negate the treatment effect and add to the duodenal obstruction. Anchoring the stent is therefore important. In the present case, the stent was fixated by OTSC clips. A small case series on different indications for OTSC clips in the gastro-intestinal tract, did not present any clip-associated adverse effects, such as stent migration, perforation, bleeding, or infections [18]. In a study overlooking 15 cases of malignant gastric outlet obstruction, treated with endoscopic stent-placement secured by OTSC clips, clinical success was achieved in 86,7 % of patients, while stent migration occurred in only 6,7 % [19]. Overall, the results suggest this method is safe and feasible. A recent alternative to OTSC clips is endoscopic suturing of the proximal end of the stent, described with promising results, but still considered technically challenging [11].

In the present case, the procedure underwent with success, with symptom relief and consistent bowel movements achieved after re-establishment of a regular diet. However, intermittent periods of diarrhea were still present, despite correct positioning of the stent. We hypothesize this was caused by retrograde passage of bowel contents beyond the distal end of the stent. This might have been avoided by primary insertion of a longer stent. A second stent-in-stent was suggested to the patient, who rejected further surgical treatment.

## 4 Conclusion

This case report demonstrated a successful endoscopic duodenal stent placement, as a palliative treatment strategy for a malignant duodenocolic fistula in an advanced colon cancer patient. Despite correct stent placement, occasional symptom recurrence was experienced, possibly due to retrograde passage of bowel contents beyond the distal end of the stent. Given the rarity of this condition, it is crucial to evaluate treatment strategies on an individual basis and in close consultation with the patient to determine the most appropriate course of action.

## Consent

Written informed consent was obtained from the patient for publication of this case report and accompanying images. A copy of the written consent is available for review by the Editor-in-Chief of this journal on request.

## Ethical approval

Written informed consent was obtained from the patient for publication of this case report and accompanying images. A copy of the written consent is available for review by the Editor-in-Chief of this journal on request.

Ethical approval is not required from the Danish Council of Ethics for publication of anonymised case reports, regarding treatment with non-experimental treatment.

## Guarantor

Claudia Jaensch.

## Sources of funding

No funding was provided for the completion of this manuscript.

## Author contribution

Study concept or design: Jaensch C.

Data collection: Bjørnå G, Jaensch C, Ørntoft MW.

Data Analysis or Interpretation: Bjørnå G, Jaensch C, Ørntoft MW.

Writing – Original Draft: Bjørnå G.

Writing – Review and Editing: Jaensch C and Ørntoft MW.

## Declaration of competing interest

The authors have no conflicts of interest to declare.
